# Clinical management and outcomes of acute febrile illness in children attending a tertiary hospital in southern Ethiopia

**DOI:** 10.1186/s12879-022-07424-0

**Published:** 2022-05-04

**Authors:** Techalew Shimelis, Susana Vaz Nery, Birkneh Tilahun Tadesse, Adam W. Bartlett, Fitsum W/Gebriel Belay, Gill Schierhout, Sabine Dittrich, John A. Crump, John M. Kaldor

**Affiliations:** 1 Kirby Institute, University of New South Wales, Sydney, Australia; 2 College of Medicine and Health Sciences, Hawassa University, Hawassa, Ethiopia; 3 The George Institute for Global Health, University of New South Wales, Sydney, Australia; 4 Foundation for Innovative New Diagnostics, Geneva, Switzerland; 5 Nuffield Department of Medicine, University of Oxford, Oxford, UK; 6 Centre for International Health, University of Otago, Dunedin, New Zealand

**Keywords:** Fever, Pneumonia, Antibacterial, Antimalarial, Overprescribing, Clinical management, Outcome

## Abstract

**Background:**

The management of febrile illnesses is challenging in settings where diagnostic laboratory facilities are limited, and there are few published longitudinal data on children presenting with fever in such settings. We have previously conducted the first comprehensive study of infectious aetiologies of febrile children presenting to a tertiary care facility in Ethiopia. We now report on clinicians’ prescribing adherence with guidelines and outcomes of management in this cohort.

**Methods:**

We consecutively enrolled febrile children aged 2 months and under 13 years, who were then managed by clinicians based on presentation and available laboratory and radiologic findings on day of enrolment. We prospectively collected outcome data on days 7 and 14, and retrospectively evaluated prescribing adherence with national clinical management guidelines.

**Results:**

Of 433 children enrolled, the most common presenting syndromes were pneumonia and acute diarrhoea, diagnosed in 177 (40.9%) and 82 (18.9%), respectively. Antibacterial agents were prescribed to 360 (84.7%) of 425 children, including 36 (34.0%) of 106 children without an initial indication for antibacterials according to guidelines. Antimalarial drugs were prescribed to 47 (11.1%) of 425 children, including 30 (7.3%) of 411 children with negative malaria microscopy. Fever had resolved in 357 (89.7%) of 398 children assessed at day 7, and in-hospital death within 7 days occurred in 9 (5.9%) of 153 admitted patients. Among children with pneumonia, independent predictors of persisting fever or death by 7 days were young age and underweight for age. Antibacterial prescribing in the absence of a guideline-specified indication (overprescribing) was more likely among infants and those without tachypnea, while overprescribing antimalarials was associated with older age, anaemia, absence of cough, and higher fevers.

**Conclusion:**

Our study underscores the need for improving diagnostic support to properly guide management decisions and enhance adherence by clinicians to treatment guidelines.

**Supplementary Information:**

The online version contains supplementary material available at 10.1186/s12879-022-07424-0.

## Background

Mortality rates in children under 5 years old have fallen over the past 3 decades [1, 2], but remain high in many resource-limited settings [3]. Most child deaths are associated with infection-related illnesses that commonly present with fever and should be treatable with simple and affordable existing interventions [1]. Hence, optimising case management of febrile illness is key to improving child survival [4].

The expanded use of rapid diagnostic tests (RDTs) for malaria has improved case management and reduced inappropriate treatment [5, 6], but there is an ongoing lack of diagnostic tools for non-malarial fever in resource-constrained settings. Integrated management guidelines developed for these settings are therefore largely dependent on clinical diagnosis with its well-recognised limitations [7, 8]. Use of these guidelines has promoted early treatment of pneumonia [9], improved patient outcomes [10], and reduced healthcare cost [11], but may also lead to both overprescription and missed treatment opportunities [12–14].

Further to the inherent limitations of empiric guidelines, healthcare workers may make decisions that deviate from guidelines and further contribute to inappropriate management of febrile illnesses. For example, in the absence of severe clinical signs, antimicrobial therapy (antibacterial/antimalarial agents) is not always necessary for fever with no apparent source of infection, and planning follow-up monitoring without initiating these drugs are recommended [7]. However antimicrobials may be prescribed unnecessarily due to various patient- and healthcare worker-related factors [15, 16], particularly for patients with a negative malaria test result [5]. In addition to generating adverse clinical outcomes, inappropriate management of febrile illness wastes healthcare resources, reduces confidence in the health system and enhances the development of drug resistance [17]. More information is therefore needed to understand prescribing practice, particularly in settings where diagnostic facilities remain limited.

Ethiopia has seen substantial reductions in under-five year old mortality [18], largely due to reductions in malaria [19] and pneumonia [20], and has set ambitious targets for further improvements [21]. In this regard, understanding factors which determine outcome of major childhood illness is important to help identify those with a higher risk of poorer outcomes so that appropriate intervention measures can be considered. We recently reported on common pathogens and their antimicrobial susceptibility patterns in children with fever attending a large tertiary hospital in the south of the country [22]. Within the same cohort, we conducted an evaluation, the first in Ethiopia and one of very few in Africa, of clinicians’ adherence to guidelines in prescribing antibacterials and antimalarials, and analysed clinical outcomes at two weeks follow-up. We evaluated whether withholding antimicrobials following guidelines is a safe management approach to reduce unnecessary use without compromising the clinical outcome. We also assessed predictors of hospitalization and outcomes among children with pneumonia, the leading cause of child mortality and hospital admissions [20].

## Methods

### Study design and setting

This study is reported as per the Strengthening the Reporting of Observational Studies in Epidemiology (STROBE) guideline (Additional file 1: Checklist S1). As described previously [22], children with fever were prospectively enrolled from May 2018 through February 2019 at Hawassa University Comprehensive Specialized Hospital (HUCSH) in southern Ethiopia. The original prospective protocol had been designed to assess common pathogens and outcomes of fever. However, results from an initial analysis of our dataset led to inclusion of analysis of predictors of hospitalization and outcomes among children with pneumonia. A retrospective evaluation of clinicians’ management practice, including antibacterial and antimalarial prescribing, in relation to guidelines and clinical outcomes was also added to inform on needed improvement of management approaches. With a 450-bed capacity, HUCSH is the largest tertiary level public health facility in the Southern Nations and Nationalities Peoples’ Region (SNNPR), and serves a large population living in SNNPR and the neighbouring region of Oromia. Children can present to HUSCH with or without a referral from a lower-level healthcare facility. Children receiving follow-up care for various infectious and non-infectious conditions can also attend scheduled visits to HUCSH. About 65% of the population in SNNPR live in areas still classified as malaria-endemic, despite recent declines in burden [23]. The routine childhood immunization schedule in Ethiopia includes vaccines for tuberculosis, diphtheria, pertussis, tetanus, poliomyelitis, measles, hepatitis B virus, *Haemophilus influenzae* type b, *Streptococcus pneumoniae*, and rotavirus, all administered within the first 12 months of birth [24].

### Study population

Study participants were children presenting to the paediatric outpatient department of HUCSH during weekday working hours, aged at least 2 months and less than 13 years with fever, defined as axillary temperature at least 37.5ºC or history of fever in the preceding 48 h, for no longer than the past 7 days. Due to ethical reasons, critically sick patients for whom blood or urine cultures were not required as part of their care at admission, were excluded. Other exclusions were as previously described [22]. Informed written consent was obtained from caregivers and assent was additionally sought from children aged 12 years.

### Data collection

#### Clinical and laboratory investigations

Clinical and laboratory data described below were captured by study staff using study-specific case report forms. Attending clinicians performed history-taking and physical examination as per the usual hospital practice. Laboratory investigations, some already routine but not universal at the hospital, and some specific to the study, were as previously described in detail [22]. Under routine hospital practice, laboratory analyses including malaria microscopy, complete blood count (CBC), human immunodeficiency virus (HIV) testing, stool microscopy, and urinalysis (both dipstick and microscopy), were available at the discretion of the clinician based on case presentation [22]. Blood, urine, and stool cultures were available for hospitalized patients when indicated by the managing clinician. Chest radiography (CXR) was performed in accordance with national guidelines when radiology services were available. Study-specific investigations undertaken for every participant regardless of presenting condition or clinician decisions were malaria microscopy, CBC, HIV testing, urinalysis, and blood and urine cultures. Blood smear microscopy is considered as the gold standard for malaria diagnosis [25, 26], and, in Ethiopia, malaria RDTs are recommended only in rural settings where microscopy is unavailable. For participants presenting with respiratory illnesses, urine was tested for *Streptococcus pneumoniae* antigen [27] via Alere BinaxNOW® *S. pneumoniae* antigen RDT (Alere Scarborough Inc, USA) to understand the significance of a urine-based pneumococcal test in guiding the diagnosis and treatment of pneumonia.

#### Diagnosis and management

Participants’ laboratory results, except for culture, were available to attending clinicians on the day of enrolment. Children were admitted based on the decision of attending clinicians. Initial treatment including antibacterial and antimalarial drugs were recorded by the study team. Participants not admitted were asked to return for follow-up assessment and management as needed on day 7 (± 1) based on clinical findings and culture results. For the purposes of the study, clinical conditions including acute diarrhoea, pneumonia, tonsillopharyngitis, and meningitis were defined (see Table 1) based on the Ministry of Health’s national guidelines for the management of common illness in hospitals [28], adopted from World Health Organization (WHO) guidelines for the management of common childhood illnesses, 2013 [8]. Laboratory confirmation was required for study-specific definition of malaria, bloodstream infections, urinary tract infections (UTIs), and anaemia (Table 1). Febrile participants in whom the source of infection was not identified based on available clinical and laboratory investigations were classified as having undifferentiated fever.

**Table 1:** Study definitions

Clinical signs	
Tachycardia	A high pulse rate for age (age: 2-11 m, > 160 beats/min; 12-47 m, > 130 beats/min; 48 m-5y, > 120 beats/min; 6-8y, > 115 beats/min; 9-12y, > 110 beats/min) [28]
Tachypnea	A high respiratory rate for age (age: 2-11 m, ≥ 50 breaths/min; 12-59 m, ≥ 40 breaths/min; 5-12y, ≥ 30 breaths/min) [28]
Illnesses	
Acute respiratory infection	Presentation with at least one respiratory sign or symptom of less than 14 days and localized to the respiratory tract (upper or lower)
Pneumonia	A history of cough and/or difficult breathing, plus sign of (a) tachypnea OR (b) chest findings OR (c) auscultatory findings OR (d) radiologic findings [28]
Acute tonsillopharyngitis	Presentation with (a) pharyngeal redness and enlarged tonsils or (b) neck lymph node and enlarged tonsils or (c) tonsillar exudate, which are suggestive of bacterial infection based on the national guidelines [28]
Unspecified upper respiratory tract infections	Presentation with at least one respiratory sign or symptom (e.g. cough, rhinorrhoea) in the absence of features consistent with other specified respiratory illnesses
Acute diarrhoea	Presentation with diarrhoea (stool frequency > 3 loose or liquid stools per day on at least one day in the week prior to enrolment) lasting less than 14 days [28]
Meningitis	Presentation with stiff neck, positive meningeal signs, or findings on cerebrospinal fluid analysis and diagnosed by clinicians as a case of meningitis [28]
Sepsis	The presence of systemic inflammation response syndrome with suspected or proven infection, or with some form of organ dysfunction [29]
Anaemia	A low haematocrit value for age (age: 2 m, < 28%; 3-6 m, < 29%; 7-24 m, < 33%; 25 m-6y, < 34%; 7-12y, < 35%) [30]
Malaria	A positive blood smear microscopy for asexual stage of *Plasmodium* species
Bloodstream infections (bacteraemia/candidaemia)	A positive blood culture for pathogenic bacteria/yeast cells
Urinary tract infection	Urine culture showing a significant bacteriuria (≥ 10^5^ and ≥ 10^4^ colony-forming-unit/ml of urine collected by clean catch and urethral catheterization, respectively) [31]
Undifferentiated fever	Cases with no identified source of infection for the fever on clinical and laboratory investigations conducted
Outcomes	
Resolved fever	Absence of fever for 2 consecutive days prior to day 7(± 1) / day 14(± 1) as reported by caregivers or measured temperature of 36.4ºC -37.5ºC
Persisting fever	Fever episode within 2 days prior to day 7(± 1) / day 14(± 1) as reported by caregivers or measured temperature of ≥ 37.5ºC
Relapsed fever	Fever reported at day 14(± 1) in a child who had resolved fever at day 7(± 1), potentially linked to the initial febrile illness
Hospitalization	Admission to hospital for treatment in relation to the presenting febrile illness
Death	Mortality within 14(± 1) days follow-up period, and potentially linked to the initial febrile illness as judged by attending clinicians

#### Evaluation of clinicians’ prescribing adherence with management guidelines

Following completion of data collection, two senior paediatricians at HUCSH, who had not been involved in clinical management of participants, reviewed every child’s study records and evaluated whether or not antibacterial agents (other than topical agents) and antimalarial drugs had been required based on guidelines for the diagnosed illnesses at initial management. On the basis of this joint review by the two paediatricians, the prescribing of antimicrobials in the absence of a guideline-specified indication is defined as overprescribing. Common diagnoses considered to be indications for antibacterial treatment according to national management guidelines [28] are presented in Additional file 2: Table S2. Considering empiric guidelines that heavily relay on clinical diagnosis and allow room for clinicians’ discretion, our approaches for evaluating adherence of prescribing antibacterial agents are as follows: (i) as cultures were not part of routine investigations in the hospital and results were not available at initial management on the day of enrolment, we took account of the clinical presentation and initial laboratory findings (faecal pus cells/red blood cells, urinalysis findings, or blood leukocytosis), and the severity of illness based on the requirement for hospital admission. (ii) Clinicians’ discretion to withhold antibacterial agents, irrespective of initial laboratory indications, but treated after confirmation of bacterial infection on culture was described as “treated on follow-up”. Prescribing antimalarial agents to children with negative malaria microscopy results was defined as overprescribing.

#### Follow-up assessment and study outcomes

Study staff gathered outcome data from interviewing the participants’ caregivers and reviewing hospital records using a study-specific follow-up form. Assessed outcomes included resolution or persistence of fever; relapse of fever; unscheduled re-consultation at the same health facility or other facilities; length of stay in hospital, hospitalization at follow-up, and death during the period. Outcome data for children not admitted were collected during the scheduled hospital visit on day 7 (± 1) or by telephone interview with caregivers if they had not returned. Hospital records of inpatients were reviewed to capture outcome data. All caregivers were contacted by telephone on day 14 (± 1) for the final follow-up assessment.

### Data analysis

Data entry and analysis were performed using SPSS version 20 (IBM Corp., New York, USA). The WHO AnthroPlus software [32] was used to compute anthropometric z-scores. Children with weight-for-age, height-for-age, and body-mass-index-for-age z-scores of less than -2 were classified as underweight, stunting, and wasting, respectively, while z-scores of at least -2 were defined to be in the normal range. Cases with missing values were excluded, and analyses were based on the number of non-missing values*.* Frequencies (with percentages) were used to summarize results of demographic and clinical characteristics, diagnoses and treatment, and the outcome at follow-up. Medians (interquartile range, IQR) were calculated to summarize results of continuous variables including age, duration of fever, and hospital stay. Difference between proportions was assessed using chi-square test. Logistic regression analyses were performed to determine crude odds ratio (COR) for initial assessment of factors associated with the following outcomes: (i) antibacterial or antimalarial overprescription; (ii) hospital admission and (iii) persisting fever or death at 7 days among children diagnosed with pneumonia. Variables that showed a significant association in initial analysis were considered for multivariable logistic regression analysis to compute adjusted odds ratios (AORs). A p-value < 0.05 was considered as showing a significant association.

## Results

### Demographic and clinical characteristics of study participants

**Fig. 1:**
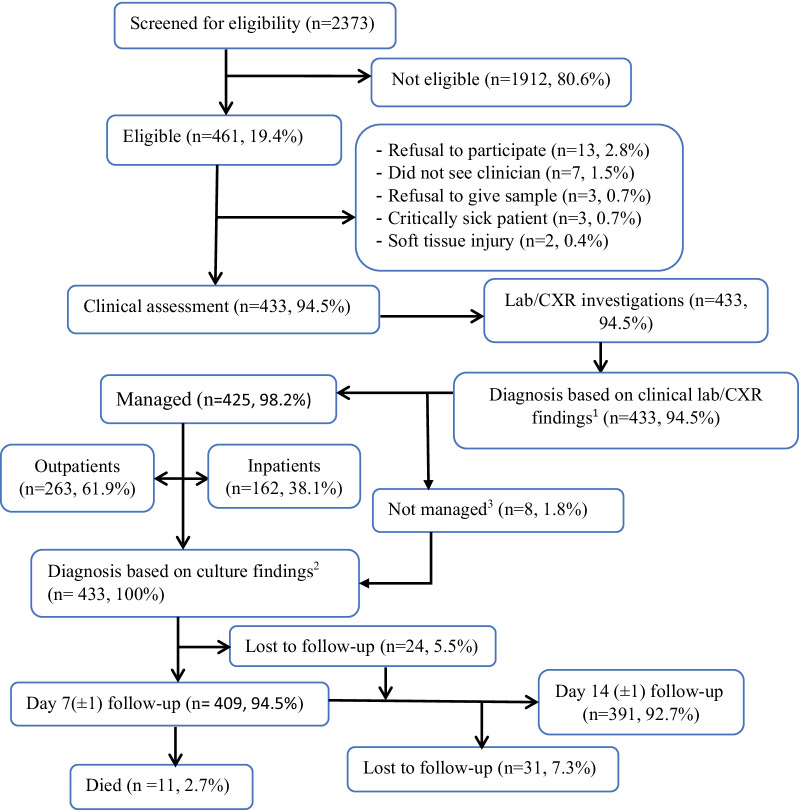
Flow diagram of patients through the study at HUCSH, 2018–2019. ^1^Diagnosis made at enrolment based on clinical features and laboratory investigations (malaria smear microscopy, complete blood count, urinalysis, stool microscopy). ^2^Diagnosis made on day 7 following release of culture results (cultures of blood, urine, stool, cerebrospinal fluid). ^3^Children left hospital without being managed. CXR, chest radiography; Lab, laboratory

The enrolment and demographic and clinical characteristics of participants have been reported in detail elsewhere [22]. Of 2,373 screened children, 461 (19.4%) met eligibility criteria during the study period, and 433 (93.9%) of these children participated with caregiver consent (Fig. 1). Twenty-eight (6.1%) of eligible children did not participate; 3 (0.7%) were excluded being critically ill. The median (IQR) age of participants was 20 (9.5–48.0) months, and 178 (41.1%) were female.

### Diagnosis

Among the 433 participants, 274 (63.3%) were classified as meeting the study definition of acute respiratory infection (ARI), as shown in Table 2. Pneumonia was the most frequent ARI, diagnosed in 177 (40.9%) of 433 participants, based on either CXR in 54 (64.3%) of 84 with CXR examinations, or reported symptoms and signs. Among 173 children with pneumonia from whom blood culture was obtained, 15 (8.7%) had bacteraemia. Among 182 children with ARIs tested for *S. pneumoniae* urine antigen, 22 (21.4%) of 103 with pneumonia were positive, compared to 9 (11.4%) of 79 diagnosed with other ARIs (p = 0.076).

**Table 2:** Infectious conditions diagnosed in febrile children attending HUCSH, 2018–2019

Infectious conditions diagnosed*	Frequency (%) (N = 433)
Acute respiratory infections	274 (63.3)
Tonsillopharyngitis	45 (10.4)
Unspecified URTIs	52 (12.0)
Pneumonia	177 (40.9)
Other LRTIs	7 (1.6)
Acute diarrhoea	82 (18.9)
Meningitis	10 (2.3)
Infective endocarditis	6 (1.4)
Otitis media	5 (1.2)
Sepsis^$^	5 (1.2)
Disseminated tuberculosis	4 (0.9)
Tinea capitis/corporis	3 (0.7)
Lymphadenitis	2 (0.5)
Bacterial conjunctivitis	2 (0.5)
Pyomyositis	2 (0.5)
Superinfected haemangioma	2 (0.5)
Malaria^†^	14 (3.2)^a^
HIV infection^†^	3 (0.7)^a^
Bacteraemia/candidaemia^ℼ^	27 (6.4)^b^
Urinary tract infection^ℼ^	74 (18.4)^c^
Other infectious conditions^‡^	5 (1.2)
Undifferentiated fever^#^	61 (14.1)

*URTIs* upper respiratory tract infections, *LRTIs* lower respiratory infections, *HIV* human immunodeficiency virus

*Diagnoses made based on clinical investigations at initial management unless otherwise specified

Children from whom samples were obtained for blood smear microscopy and HIV testing: ^a^(N = 431); blood culture ^b^(N = 421); urine culture ^c^(N = 402)

^**†**^Diagnosis made at presentation based on laboratory investigations

^ℼ^Diagnosis made on follow-up based on culture results

^‡^Spontaneous bacterial peritonitis (n = 1), neck abscess (n = 1), mumps (n = 1), appendiceal mass (n = 1), scabies (n = 1)

^$^Sepsis with bacteraemia (n = 2), sepsis without bacteraemia (n = 1), no adequate blood sample for culture (n = 2)

^#^Cases with no identified source of infection for the fever on clinical and laboratory investigations conducted

Of 433 children, 82 (18.9%) had acute diarrhoea. A UTI was detected in 74 (18.4%) of 402 children [22]. At presentation, 75 (17.3%) of 433 children had non-specific fever (without localizing signs or symptoms on clinical examination); of these, 14 (18.7%) had a UTI, and 3 (4.0%) had bacteraemia. A number of non-infectious conditions were also diagnosed among the participants (Additional file 3: Table S3).

### Clinical management

#### Hospitalization on initial management

On the day of enrolment, 425 (98.2%) of 433 children were managed based on available clinical, laboratory, and radiologic findings, while 8 (1.8%) children left the hospital before care was provided. Of 425 children, 162 (38.1%) were admitted as inpatients, and 263 (61.9%) were managed as outpatients. Of children admitted, 101 (62.3%) were classified as having pneumonia.

#### Antibacterial and antimalarial prescriptions and adherence with guidelines

As shown in Table 3, antibacterial agents were prescribed to 360 (84.7%) of 425 children, 355 (85.5%) of 425 on initial management. Among inpatients, 151 (93.2%) of 162 received antibacterial agents compared to 204 (77.6%) of 263 outpatients. Of 425 children, 106 (24.9%) were judged retrospectively not to have had an initial indication for antibacterial treatment; 36 (34.0%) of 106 children without an initial indication were overprescribed antibacterials. Of 319 children judged to have had indication for antibacterial therapy, 41 (12.9%) were based on initial laboratory findings suggestive of infections (faecal pus cell/red blood cell, urinalysis findings, or blood leukocytosis) although bacteria were not found on cultures. Antimalarial agents were prescribed to 47 (11.1%) of 425 children; 30 (7.3%) of 411 children with negative malaria microscopy were overprescribed antimalarials; of which, 12 were inpatients and 18 were outpatients. A further 3 inpatients with persisting fever on follow-up were also treated empirically for malaria, despite a negative initial and follow-up test. Antibacterial and antimalarial agents were overprescribed in 12 (33.3%) of 36 and 13 (43.3%) of 30 children with undifferentiated fever, respectively.

**Table 3:** Antibacterial and antimalarial prescriptions and adherence with guidelines in febrile children attending HUCSH, 2018–2019

Prescriptions and adherence with guidelines	Antibacterial treatment n (%) N = 425	Antimalarial treatment n (%) N = 425
Total children prescribed	360 (84.7)	47 (11.1)
With indication and prescribed on initial management^1^	319 (75.0)	14 (3.3)
Overprescribed on initial management^2^	36 (8.5)	30 (7.1)
Overprescribed among children without indication	36 (34.0)^a^	30 (7.3)^b^
Treated on follow-up^3^	5 (1.2)	-
Overprescribed on follow-up^4^	-	3 (0.7)
Total children not prescribed	65 (15.3)	378 (88.9)
Without indication and not prescribed ^5^	56 (13.2)	-
With indication and not prescribed ^6^	9 (2.1)	-

^a^(N = 106), ^b^(N = 411)

^1^Prescribed on initial management as suggested by the national management guideline, irrespective of culture findings on follow-up

^2^Prescribed on initial management without enough clinical and/or initial laboratory indications according to the guideline and no pathogen was identified on culture as well

^3^Not prescribed on initial management (irrespective of initial indications) but treated after confirmation of bacterial infection on culture

^4^Prescribed to cases with persisting fever despite malaria negative result on follow-up

^5^Not prescribed on initial management as suggested by the guideline and no pathogen was identified on culture as well

^6^Not prescribed on initial management or follow-up at the hospital despite enough indications due to clinician discretion or lost to follow-up

### Outcomes

Outcome results on days 7 and 14 are presented in Table 4. Day 7 (± 1) follow-up data were gathered from 409 (94.5%) of 433 participants, with 24 (5.5%) lost to follow-up because they had not returned to hospital on the scheduled date and the attempt to contact by telephone was unsuccessful. Of 409 children contacted on day 7, 153 (37.4%) had been admitted on initial management with median (IQR) stay of 5 (3–9) days. Two children had been admitted subsequent to initial assessment, one due to UTI and the other suspected malaria.

**Table 4:** Follow-up data in febrile children attending HUCSH, 2018–2019

	Day 7(± 1) follow-up n (%)	Day 14(± 1) follow-up n (%)
Follow-up status	N = 433	N = 422
Completed	409 (94.5)	391 (92.7)
Lost to follow-up	24 (5.5)	31 (7.3)
Child status	N = 409	N = 391
Live	398 (97.3)	391 (100)
Died	11 (2.7)	0
Fever status	N = 398	N = 391
Resolved	357 (89.7)	358 (91.6)
Persisted	41 (10.3)	16 (4.1)
Relapsed	-	17 (4.3)
Caregivers’ further action after first visit	N = 398	N = 391
No further action taken	371 (93.2)	380 (97.2)
Visited other facilities	19 (4.8)	7 (1.8)
Self-prescribed drug	3 (0.8)	2 (0.5)
Unscheduled re-consultation	3 (0.8)	1 (0.3)
Other	2 (0.5)	1 (0.3)
Time of hospitalization	N = 409	
Hospitalized on first visit	153 (37.4)	-
Hospitalized on follow-up	1 (0.2)	1
Not hospitalized	255 (62.3)	-

Of 409 children whose outcome was known on the day 7 contact, 11 (2.7%) had died. Of these, 5 (45.6%) had pneumonia, 2 (18.2%) sepsis, 1 (9.1%) meningitis, and 1 (9.1%) respiratory failure associated with Guillain–Barre syndrome. Other conditions diagnosed in children who died were underweight (n = 6), heart disease (n = 2), liver failure (n = 1), and anaemia (n = 3). All deaths occurred among the 153 patients who were initially hospitalized and whose outcome was known; 9 (5.9%) during hospital stay within 4 days, and 2 (1.3%) at home after leaving hospital based on caregivers’ decisions.

Fever had resolved in 357 (89.7%) of 398 surviving children by day 7, in median (IQR) 2 (1–3) days. Among non-hospitalized children without an indication for antibacterial therapy on initial management, fever had resolved by day 7 in 32 (97.0%) of 33 prescribed antibacterial agents and 47 (95.9%) of 49 children not prescribed with these drugs. Among malaria negative non-hospitalized patients, fever resolved in 17 (94.4%) of 18 children treated with antimalarial therapy compared to 213 (95.5%) of 223 patients who were not. Following initial management at the hospital, 3 (6.1%) of 49 with no antibacterial therapy and 12 (5.4%) of 223 with no antimalarial therapy reported to have sought further care from another or the same facility or taking self-prescribed drug. Persisting fever was reported by day 14 in 16 (4.1%) of 391 children, and a fever reported at 7 days as resolved had relapsed at 14 days in 17 (4.3%), mainly among those initially diagnosed with pneumonia (n = 14).

### Predictors of hospitalization, overprescribing, or persisting fever/death

Among 175 children classified as having pneumonia who received care at initial management (Additional file 4: Table S4), a higher proportion of hospital admissions was observed among those coming from outside Hawassa City (but within SNNPR) compared to those from Hawassa (AOR 3.45; 95% CI 1.03–11.6). In the same population, children with signs of chest indrawing or retraction (AOR 10.9; 95% CI 4.71–25.4) at initial management had higher odds of hospitalization compared to children with no such signs, as did those with wasting (AOR 3.86; 95% CI 1.57–9.51).

Among 106 children without an initial indication for antibacterial treatment, overprescribing antibacterials was less frequent in children aged 36–59 months (AOR 0.14; 95% CI 0.03–0.64) at initial management compared to those aged 2–11 months. Children with tachypnea (AOR 0.31; 95% CI 0.11–0.89) were also less frequently overprescribed antibacterial drugs compared to those with a normal respiratory rate (Additional file 5: Table S5). Among 411 children with negative malaria microscopy, overprescribing of antimalarials was more frequent in children aged 36–59 months (AOR 11.3; 95% CI 2.24–57.4) compared to children aged 2–11 months. A higher proportion of children with anaemia (AOR 3.45; 95% CI 1.20–9.89) were overprescribed antimalarial drugs compared to those without anaemia. Children with cough (AOR 0.29; 95% CI 0.12–0.72) were less likely overprescribed antimalarial drugs, as were those with axillary temperature under 37.5ºC (AOR 0.05; 95% CI 0.01–0.46) or 37.5–38.9ºC (AOR 0.19; 95% CI 0.08–0.48) compared to children with temperature above 39ºC (Additional file 6: Table S6).

Among 168 children with pneumonia whose outcomes were assessed at day 7, those aged 12–35 months (AOR 0.16; 95% CI 0.03–0.76) or aged at least 36 months (AOR 0.07; 95% CI 0.01–0.76) were less likely to have persisting fever or to have died compared with those aged 2–11 months. Further, the odds of having persistent fever or death were lower among those with vomiting (AOR 0.21; 95% CI 0.05–0.79) compared to those without. A higher proportion of pneumonia patients classified as underweight (AOR 3.63; 95% CI 1.14–11.6) had persisting fever or had died compared to those with normal weight-for-age z-scores (Additional file 7: Table S7).

## Discussion

In this comprehensive analysis of clinical management and outcome of febrile illnesses, we identified overprescription of antibacterial and antimalarial agents as a key issue. Specifically, antibacterial agents were overprescribed to 34.0% of children without an initial indication for treatment based on the national guidelines. We also found that 7.3% of children with negative malaria microscopy were overprescribed antimalarial drugs on initial management. Overprescribing antibacterial agents occurred more in younger children, and in those without tachypnea. Overprescribing antimalarials was independently predicted by older age, anaemia, absence of cough and higher fever at presentation. Independent predictors of persisting fever or death among children with pneumonia were age under 12 months, absence of vomiting, and being underweight. Among children whose conditions were determined not to have met guidelines for antibacterial or antimalarial treatment, there was no difference in outcome between those who did and did not receive these agents.

Our study has the strength of being the first in Ethiopia to assess clinical management of childhood febrile illnesses against guidelines and in light of clinical outcomes. Also, there have been very few studies from African countries that reported on management and outcomes of febrile illnesses. Results from this evaluation should inform management approaches and adherence of health workers to policy recommendations of prescribing and withholding antibacterial and antimalarial agents. However, interpretation of our findings needs to take account of several limitations to our study design. First, our goal of investigating fever aetiology in the same cohort [22] introduced laboratory investigations which went beyond routine practice in the hospital, potentially influencing clinicians’ practice. Second, our laboratory and other diagnostic procedures did not cover all known infectious diseases, limiting our ability to inform on needed improvement of fever management guidelines. Third, missing data due to absence of specimens and loss to follow-up in some children might have introduced bias. Fourth, given that clinicians’ treatment decisions are influenced by various factors, including patients’ clinical presentation and treatment history, availability of drugs, known antimicrobial resistance patterns, and cost, we focussed our evaluation on broad categories of treatment. Last, there may have been recall and other errors in information gained from caregivers’ self-report.

Pneumonia remained the leading syndrome associated with fever presenting at this tertiary hospital despite a reduction in incidence and mortality due to implementation of interventions in Ethiopia including vaccination against *Streptococcus pneumoniae* and *Haemophilus influenzae* type b [20]. Ongoing dependence on relatively non-specific clinical case definitions that prioritise sensitivity [28] in the absence of applicable diagnostic tools and limited access to radiography hinder efforts to curb overuse of antibacterial agents. Blood culture is available to support clinical decision making but it involves a time delay and is generally considered to have a yield of bacteraemia in pneumonia of less than 10% [33], a finding that we replicated (8.7%) as have others in Ethiopia (5.6%) [34]. Urine-based pneumococcal tests are also of limited accuracy [35, 36] in guiding treatment due to pneumococcal carriage, resulting in urinary excretion [37], false positivity following resolved infection, and cross reaction with other closely related streptococci [38, 39]. We also observed no significant difference in proportions of children positive for urine *S. pneumoniae* antigen by pneumonia status, as other investigators found [14]. There remains an urgent need for rapid and inexpensive tests that can differentiate bacterial from non-bacterial causes of respiratory infections, including pneumonia and tonsilopharyngitis to guide clinical decision-making and optimal use of antibacterial agents.

Prescribing antibacterial agents based on presence of faecal pus cells/red blood cells, as suggested by the guideline for dysentery, may also lead to over-prescription in settings where enteric bacterial infections such as shigellosis and salmonellosis are rare [22]. UTI also poses particular difficulties due to the non-specific nature of symptoms, and requires providing routine screening with urinalysis for febrile children as commonly practised in high income countries [31].

The proportion of overprescribing antibacterial agents in the present study (34.0%) was lower than that reported from a study in Uganda (42.0%) [15]. Further, our finding about overprescribing antimalarial drugs to patients with negative malaria test (7.1%) was comparable to a result in Tanzania (11.5%) [40] although higher proportions of overprescribing (21.0–58.0%) [41, 42] were reported from other African countries. A lower odds of overprescribing antibacterial treatment in older children compared to infants might be due to the likelihood of more conservative empiric antibacterial treatment for infants. The lower odds of overprescribing of antibacterial agents for those with tachypnoea may be because it is a sign of pneumonia. For antimalarials, overprescribing was more common in older children, as it was in children with anaemia, with higher fever, or without cough, clinical characteristics that clinicians may associate with malaria to support a decision to undertake empiric treatment. Notably, antibacterial and antimalarial agents were frequently overprescribed to children with undifferentiated fever. This may suggest that the lack of diagnostic tests for non-malarial fever influences the prescription of both types of drug, as long as the source of fever remains unexplained.

A better understanding of clinician non-adherence to treatment guidelines will benefit from qualitative research into motivating factors. Clinicians may be simply covering themselves against the possibility of missed diagnoses and consequent disease progression if children are sent home with no medication. We found that the proportion of participants without indications for antibacterial or antimalarial therapy who recovered at 7 days was not affected by whether or not they actually received therapy, consistent with earlier findings in relation to malaria treatment in the presence of a negative RDT result [12, 43]. This finding might provide reassurance to clinicians facing this situation, and support a more rational approach based on ensuring adequate follow-up monitoring in circumstances where it is feasible. Uncertainty about the quality of malaria microscopy may have prompted empiric treatment, potentially suggesting a need for strengthening laboratory quality assurance program to enhance confidence of clinicians on laboratory findings. In this regard, re-testing with malaria RDTs may be helpful where microscopy is negative but there is a high index of suspicion, and provide further support for clinical decision-making within the guidelines. The adherence of caregivers to clinicians’ management advice, particularly in children sent home with no antimalarial or antibacterial prescription, is important to efforts of promoting rational use of antibacterial and antimalarial agents.

A higher proportion (38.1%) of febrile children were hospitalized in our study compared to previous reports from African countries (5.3–24.0%) [44–47], possibly because our recruitment was from a tertiary facility which may have attracted patients with more severe disease than the primary care centres that were the sites for most earlier studies. Pneumonia as a main cause of hospitalization calls for improved access for timely management to minimise disease progression. We found that children with signs of severe respiratory distress (lower chest indrawing or retraction) were more likely to require admission [8, 28]. We also saw increased odds of hospitalization among children with wasting, and poorer outcomes (persisting fever or death) among infants, who may have had incomplete immunization due to their age, and in those who were underweight. Our findings suggest the need for intensifying interventions, particularly enhancing nutritional status to reduce the risk of acquiring pneumonia and improving outcomes [48].

Overall, we found that fever had resolved in 89.7% of participants by 7 days, consistent with earlier findings from African studies of children with uncomplicated febrile illnesses [12, 14, 43]. The proportion of relapse of fever at day 14 in the present study (4.3%) was similar with a single earlier report from Tanzania (4.5%) [14]. Our observed in-hospital fatality ratio of 5.9% was consistent with findings reported from Tanzania (5.7–7.3%) [44, 49] although a lower result (1.4%) was also reported from the same country [46].

## Conclusion

We found that among febrile children presenting to a tertiary care hospital there was a lack of adherence to guidelines for antibacterial and antimalarial prescriptions. Although most children also received antibacterial prescriptions based on the management guidelines, the current, largely symptom-based approach limits opportunities to optimize the use of antibacterial agents. Thus, providing sound evidence-informed clinical guidance backed by laboratory and radiology diagnostic support, and improving adherence of health workers to management guidelines are essential.

## Supplementary Information

**Additional file 1. **STROBE statement.

**Additional file 2: Table S2. **Common diagnosis considered to be indications for antibacterial treatment according to national management guidelines [28].

**Additional file 3: Table S3.** Non-infectious conditions diagnosed in febrile children attending HUCSH, 2018-2019.

**Additional file 4: Table S4.** Predictors of hospitalization among children with pneumonia attending HUCSH, 2018-2019.

**Additional file 5: Table S5.** Predictors of overprescribing antibacterials on initial management among children attending HUCSH, 2018-2019.

**Additional file 6: Table S6. **Predictors of overprescribing antimalarials on initial management to children without confirmed malaria at HUCSH, 2018-2019

**Additional file 7: Table S7.** Predictors of persisting fever or death by day 7 (±1) among children with pneumonia at HUCSH, 2018-2019.

## Data Availability

The datasets used and/or analysed during the current study are available from the corresponding author on reasonable request.

## Abbreviations

RDT: Rapid diagnostic test
WHO: World Health Organization
UTI: Urinary tract infection
HUCSH: Hawassa University Comprehensive Specialized Hospital
SNNPR: Southern Nations and Nationalities Peoples’ Region
HIV: Human immunodeficiency virus
CBC: Complete blood count
ARI: Acute respiratory infection
URTI: Upper respiratory tract infection
IQR: Interquartile range
COR: Crude odds ratio
AOR: Adjusted odds ratio
CXR: Chest radiography
