# Supplementary material for: Clinical management and outcomes of acute febrile illness in children attending a tertiary hospital in southern Ethiopia
Source: BMC Infect Dis. 2022 May 4;22:434. doi: 10.1186/s12879-022-07424-0 (PMC9069758; doi:10.1186/s12879-022-07424-0)
Supplement: Supplementary file 2 — Additional file 2: Table S2. Common diagnosis considered to be indications for antibacterial treatment according to national management guidelines [28]. [file 12879_2022_7424_MOESM2_ESM.docx]

S2 Table: Common diagnosis considered to be indications for antibacterial treatment according to national management guidelines [26].

| Diagnosis considered to be indications for antibacterial treatment | Diagnosis considered not to be indications for antibacterial treatment^3^ |
| --- | --- |
| Tonsilopharyngitis | Upper respiratory infections (non-specified) |
| Pneumonia | Malaria |
| Lower respiratory infections (non-specified) | Intestinal parasites |
| Urinalysis findings suggestive of urinary tract infections (presence of pyuria/leucocyte esterase/nitrite)^1^ | Gastrointestinal bleeding- non infective |
| Dysentery or stool findings suggestive of enteric bacterial infections (presence of fecal pus cells and red blood cells)^1^ | Non-dysenteric diarrhea without stool findings suggestive of enteric bacterial infections |
| Blood leukocytosis suggestive of bacterial infection^1^ | Non-severe febrile illness (non-specified) |
| Meningitis | Human immunodeficiency virus^4^ |
| Sepsis | Mumps |
| Endocarditis | Fungal infections |
| Acute otitis media | Dermatitis |
| Bacterial conjunctivitis | Allergies |
| Pyogenic lymphadenitis | Anaemia |
| Abscess/infected tissue | Reactive airway disease/asthma |
| Appendiceal mass | Febrile seizure |
| Severe febrile disease (non-specified)^2^ | Flaccid paralysis |
| Acute glomerulonephritis | Scabies |
| Pyomyositis | Goiter |
| Severe acute malnutrition | Epilepsy |
| Peritonitis | Down syndrome |
| Intussusception | Cushing syndrome |
| Culture confirmed bacteraemia | Rickets |
| Culture confirmed urinary tract infection | Edwards syndrome |
| Culture confirmed shigellosis | Mild/moderate malnutrition |
| Culture confirmed salmonellosis |  |

^1^ Clinicians’ discretion to withhold antibacterial agents among these cases was not considered as non-adherence if bacterial infection was treated after confirmation on culture.

^2^ Severity of illness is based on the requirement for hospital admission.

^3^ However, antibacterial treatment is indicated if these conditions co-exist with those specified in the left-hand column.

^4^ Human immunodeficiency virus is not an indication for antibacterial treatment, but antibacterial prophylaxis may be prescribed, which is appropriate.
