# Supplementary material for: Clinical management and outcomes of acute febrile illness in children attending a tertiary hospital in southern Ethiopia
Source: BMC Infect Dis. 2022 May 4;22:434. doi: 10.1186/s12879-022-07424-0 (PMC9069758; doi:10.1186/s12879-022-07424-0)
Supplement: Supplementary file 3 — Additional file 3: Table S3. Non-infectious conditions diagnosed in febrile children attending HUCSH, 2018-2019. [file 12879_2022_7424_MOESM3_ESM.docx]

S3 Table: Non-infectious conditions diagnosed in febrile children attending HUCSH, 2018-2019

| Diagnosed non-infectious conditions | Frequency (%)  (N=433) |
| --- | --- |
| Wasting^1^ | 121 (28.0)^d^ |
| Stunting^2^ | 90 (20.8)^d^ |
| Underweight^3^ | 101 (24.1)^e^ |
| Anaemia | 51 (11.9)^f^ |
| Heart disease | 15 (3.5) |
| Reactive airway disease/asthma | 9 (2.1) |
| Rickets | 5 (1.2) |
| Intussusception | 4 (0.9) |
| Guillain-Barre Syndrome/ flaccid paralysis | 4 (0.9) |
| Acute glomerulonephritis | 3 (0.7) |
| Other non-infectious conditions^⸙^ | 20 (4.6) |

^1^Body-mass-index-for-age z-score (BAZ, < -2); ^d^(N=432)

^2^Height-for-age z-score (HAZ, < -2)

^3^Weight-for-age z-score (WAZ, < -2); WAZ was calculated only for children up to 10 years of age:  ^e^(N=419)

Children from whom blood samples were obtained for haematocrit determination: ^f^ (N=427)

**^⸙^**Atopic dermatitis (n=2), febrile seizure (n=2), Down syndrome (n=2), hematologic malignancy (n=2), hydrocephalus (n=2), microcephaly (n=1), allergic conjunctivitis (n=1), goitre (n=1), Edwards syndrome (n=1), epilepsy (n=1), brain tumour (n=1), Cushing syndrome (n=1), nephrotic-nephritic syndrome (n=1), upper gastrointestinal bleeding (n=1), liver failure (n=1)
