# Supplementary material for: Clinical management and outcomes of acute febrile illness in children attending a tertiary hospital in southern Ethiopia
Source: BMC Infect Dis. 2022 May 4;22:434. doi: 10.1186/s12879-022-07424-0 (PMC9069758; doi:10.1186/s12879-022-07424-0)
Supplement: Supplementary file 4 — Additional file 4: Table S4. Predictors of hospitalization among children with pneumonia attending HUCSH, 2018-2019. [file 12879_2022_7424_MOESM4_ESM.docx]

S4 Table: Predictors of hospitalization among children with pneumonia attending HUCSH, 2018-2019

| Characteristics | Pneumonia cases | | COR (95% CI)  (Included in the analysis, N=175) | AOR (95% CI) |
| --- | --- | --- | --- | --- |
|  | **Not hospitalized**  **n (%) N=74** | **Hospitalized**  **n (%) N=101** |  |  |
| Residence Adm. Region |  |  |  |  |
| SNNPR-Hawassa | 47 (63.5) | 39 (38.6) | 1 | 1 |
| SNNPR-other | 6 (8.1) | 22 (21.8) | **4.42 (1.63-12.0)*** | **3.45 (1.03-11.6)*** |
| Oromia | 21 (28.4) | 40 (65.6) | **2.30 (1.17-4.52)*** | 2.27 (0.96-5.35) |
| Gender |  |  |  |  |
| Male | 44 (59.5) | 58 (57.4) | 1 | - |
| Female | 30 (40.5) | 43 (42.6) | 1.09 (0.59-2.0) |  |
| Age |  |  |  |  |
| 2 - 11 m | 25 (33.8) | 47 (46.5) | 1 | 1 |
| 12 - 35 m | 29 (39.2) | 37 (36.6) | 0.68 (0.34-1.35) | 0.70 (0.29-1.68) |
| 36 - 59 m | 10 (13.5) | 11 (10.9) | 0.59 (0.22-1.57) | 0.37 (0.10-1.31) |
| 5 - 12 y | 10 (13.5) | 6 (5.9) | **0.32 (0.10-0.98)*** | 0.42 (0.10-1.68) |
| Duration of fever |  |  |  |  |
| 1 day | 15 (20.3) | 16 (15.8) | 1 |  |
| 2 - 4 days | 38 (51.4) | 63 (62.4) | 1.55 (0.69-3.50) | - |
| 5 - 7 days | 21 (28.4) | 22 (21.8) | 0.98 (0.39-2.47) |  |
| Antibacterial treatment prior to visit for current episode |  |  |  |  |
| Yes | 21 (28.4) | 31 (30.7) | 1.12 (0.58-2.16) | - |
| No | 53 (71.6) | 70 (69.3) | 1 |  |
| Vaccination status |  |  |  |  |
| Completed | 52 (70.3) | 61 (60.4) | 1 |  |
| Vaccinated for age | 18 (24.3) | 33 (32.7) | 1.56 (0.79-3.10) | - |
| Other | 4 (5.4) | 7 (6.9) | 1.49 (0.41-5.38) |  |
| Axillary temperature |  |  |  |  |
| <37.5ºC ^§^ | 8 (10.8) | 4 (4.0) | 1 | 1 |
| 37.5 - 38.9ºC | 60 (81.1) | 81 (80.2) | 2.70 (0.78-9.38) | 2.87 (0.62-13.2) |
| ≥39ºC | 6 (8.1) | 16 (15.8) | **5.33 (1.16-24.5)*** | 3.34 (0.53-21.1) |
| Vomiting |  |  |  |  |
| Yes | 21 (28.4) | 37 (36.6) | 1.46 (0.76-2.79) | - |
| No | 53 (71.6) | 64 (63.4) | 1 |  |
| Diarrhoea |  |  |  |  |
| Yes | 6 (8.1) | 18 (17.8) | 2.46 (0.92-6.54) | - |
| No | 68 (91.9) | 83 (82.2) | 1 |  |

| Grunting |  |  |  |  |
| --- | --- | --- | --- | --- |
| Yes | 10 (13.5) | 21 (20.8) | 1.68 (0.74-3.82) | - |
| No | 64 (86.5) | 80 (79.2) | 1 |  |
| Tachycardia |  |  |  |  |
| Yes | 28 (37.8) | 50 (49.5) | 1.61 (0.88-2.97) | - |
| No | 46 (62.2) | 51 (50.5) | 1 |  |
| Tachypnea |  |  |  |  |
| Yes | 61 (82.4) | 91 (90.1) | 1.94 (0.80-4.70) | - |
| No | 13 (17.6) | 10 (9.9) | 1 |  |
| Lower chest indrawing/ retraction |  |  |  |  |
| Yes | 11 (14.9) | 68 (67.3) | **11.8 (5.50-25.3)*** | **10.9 (4.71-25.4)*** |
| No | 63 (85.1) | 33 (32.7) | 1 | 1 |
| Crepitation |  |  |  |  |
| Yes | 35 (47.3) | 68 (67.3) | **2.30 (1.24-4.26)*** | 1.87 (0.83-4.20) |
| No | 39 (52.7) | 33 (32.7) | 1 | 1 |
| WBC count |  |  |  |  |
| Normal | 58 (78.4) | 67 (67.0)^a^ | 1^Ᵹ^ | - |
| High | 12 (16.2) | 23 (23.0)^a^ | 1.66 (0.76-3.63) |  |
| Low | 4 (5.4) | 10 (10.0)^a^ | 2.16 (0.64-7.27) |  |
| Anaemia |  |  |  |  |
| Yes | 4 (5.4) | 12 (12.0)^a^ | 2.39 (0.74-7.72) | - |
| No | 70 (94.6) | 88 (88.0)^a^ | 1^Ᵹ^ |  |
| WAZ |  |  |  |  |
| Normal (≥ -2) | 57 (77.0) | 64 (64.6)^b^ | 1^Ꝭ^ | - |
| Underweight (< -2) | 17 (23.0) | 35 (35.4)^b^ | 1.83 (0.93-3.62) |  |
| HAZ |  |  |  |  |
| Normal (≥ -2) | 56 (75.7) | 74 (73.3) | 1 | - |
| Stunting (< -2) | 18 (24.3) | 27 (26.7) | 1.14 (0.57-2.26) |  |
| BMI-AZ |  |  |  |  |
| Normal (≥ -2) | 61 (82.4) | 60 (59.4) | 1 | 1 |
| Wasting (< -2) | 13 (17.6) | 41 (40.6) | **3.21 (1.56-6.58)*** | **3.86 (1.57-9.51)*** |

SNNPR, Southern Nations and Nationalities Peoples’ Region, COR, crude odds ratio, AOR, adjusted odds ratio, WBC, white blood cell; WAZ, weight-for-age z-score; HAZ, height-for-age z-score; BMI-AZ, body-mass-index-for-age z-score; m, month; y, years

^a^(N=100); ^b^(N=99)

Included in the analysis; ^Ᵹ^(N=174); ^Ꝭ^(N=173)

*Significantly associated (p-value < 0.05)

**^§^** History of fever episode at least once in the preceding 48 hours
