# Supplementary material for: Clinical management and outcomes of acute febrile illness in children attending a tertiary hospital in southern Ethiopia
Source: BMC Infect Dis. 2022 May 4;22:434. doi: 10.1186/s12879-022-07424-0 (PMC9069758; doi:10.1186/s12879-022-07424-0)
Supplement: Supplementary file 5 — Additional file 5: Table S5. Predictors of overprescribing antibacterials on initial management among children attending HUCSH, 2018-2019. [file 12879_2022_7424_MOESM5_ESM.docx]

S5 Table: Predictors of overprescribing antibacterials on initial management among children attending HUCSH, 2018-2019

| Characteristics | Antibacterial treatment | | COR (95% CI)  (Included in the analysis, N=106) | AOR (95% CI) |
| --- | --- | --- | --- | --- |
|  | **Not prescribed n (%) N= 70** | **Overprescribed**  **n (%) N= 36** |  |  |
| Residence Adm. Region |  |  |  |  |
| SNNPR-Hawassa | 52 (74.3) | 24 (66.7) | 1 | - |
| SNNPR-other | 6 (8.6) | 3 (8.3) | 1.08 (0.25-4.70) |  |
| Oromia | 12 (17.1) | 9 (25.0) | 1.63 (0.60-4.38) |  |
| Gender |  |  |  |  |
| Male | 45 (64.3) | 27 (75.0) | 1 | - |
| Female | 25 (35.7) | 9 (25.0) | 0.60 (0.24-1.47) |  |
| Age |  |  |  |  |
| 2 – 11 m | 11 (15.7) | 10 (27.8) | 1 | 1 |
| 12 – 35 m | 20 (28.6) | 18 (50.0) | 0.99 (0.34-2.88) | 1.02 (0.34-3.06) |
| 36 – 59 m | 23 (32.9) | 3 (8.3) | **0.14 (0.03-0.63)*** | **0.14 (0.03-0.64)*** |
| 5 – 12 y | 16 (16.0) | 5 (13.9) | 0.34 (0.09-1.29) | 0.45 (0.11-1.76) |
| Duration of fever |  |  |  |  |
| 1 day | 25 (35.7) | 11 (30.6) | 1 | - |
| 2– 4 days | 37 (52.9) | 24 (66.7) | 1.47 (0.61-3.54) |  |
| 5– 7 days | 8 (11.4) | 1 (2.8) | 0.28 (0.03-2.56) |  |
| Antibacterial treatment prior to visit for current episode |  |  |  |  |
| Yes | 13 (18.6) | 8 (22.2) | 1.25 (0.47-3.37) | - |
| No | 57 (81.4) | 28 (77.8) | 1 |  |
| Axillary temperature |  |  |  |  |
| <37.5ºC ^§^ | 13 (18.6) | 6 (16.7) | 1.29 (0.32-5.26) | - |
| 37.5 – 38.9ºC | 43 (61.4) | 25 (69.4) | 1.63 (0.53-5.06) |  |
| ≥39ºC | 14 (20.0) | 5 (13.9) | 1 |  |
| Cough |  |  |  |  |
| Yes | 10 (14.3) | 8 (22.2) | 1.71 (0.61-4.81) | - |
| No | 60 (85.7) | 28 (77.8) | 1 |  |
| Vomiting |  |  |  |  |
| Yes | 26 (37.1) | 18 (50.0) | 1.69 (0.75-3.82) | - |
| No | 44 (62.9) | 18 (50.0) | 1 |  |
| Diarrhoea |  |  |  |  |
| Yes | 9 (12.9) | 7 (19.4) | 1.64 (0.55-4.83) | - |
| No | 61 (87.1) | 29 (80.6) | 1 |  |
| Tachypnea |  |  |  |  |
| Yes | 28 (40.0) | 6 (16.7) | **0.03 (0.11-0.81)*** | **0.31 (0.11-0.89)*** |
| No | 42 (60.0) | 30 (83.3) | 1 | 1 |
| Tachycardia |  |  |  |  |
| Yes | 20 (28.6) | 11 (30.6) | 1.10 (0.46-2.65) | - |
| No | 50 (71.4) | 25 (69.4) | 1 |  |

| Lower chest indrawing/ retraction |  |  |  |  |
| --- | --- | --- | --- | --- |
| Yes | 70 (100) | 0 (0) | - | - |
| No | 70 (100) | 36 (100) |  |  |
| Crepitation |  |  |  |  |
| Yes | 70 (100) | 0 (0) | - | - |
| No | 70 (100) | 36 (100) |  |  |
| WBC count |  |  |  |  |
| Normal | 58 (82.9) | 31 (86.1) | 1 | - |
| High | 1 (1.4) | 0 (0) | - |  |
| Low | 11 (15.7) | 5 (13.9) | 0.85 (0.27-2.67) |  |
| Anaemia |  |  |  |  |
| Yes | 12 (17.1) | 3 (8.3) | 0.44 (0.12-1.67) | - |
| No | 58 (82.9) | 33 (91.7) | 1 |  |
| WAZ |  |  |  |  |
| Normal (≥ -2) | 59 (88.1)^c^ | 29 (80.6) | 1^Ꝿ^ | - |
| Underweight (< -2) | 8 (11.9)^c^ | 7 (19.4) | 1.78 (0.59-5.39) |  |
| HAZ |  |  |  |  |
| Normal (≥ -2) | 62 (88.6) | 27 (75.0) | 1 | - |
| Stunting (< -2) | 8 (11.4) | 9 (10.2) | 2.58 (0.90-7.41) |  |
| BMI-AZ |  |  |  |  |
| Normal (≥ -2) | 53 (75.7) | 27 (75.0) | 1 | - |
| Wasting (< -2) | 17 (24.3) | 9 (25.0) | 1.04 (0.41-2.64) |  |

SNNPR, Southern Nations and Nationalities Peoples’ Region, COR, crude odds ratio, AOR, adjusted odds ratio, WBC, white blood cell; WAZ, weight-for-age z-score; HAZ, height-for-age z-score; BMI-AZ, body-mass-index-for-age z-score; m, month; y, years

^c^(N= 67); Included in the analysis; ^Ꝿ^(N=103)

*Significantly associated (p-value < 0.05)

**^§^** History of fever episode at least once in the preceding 48 hours
