# Supplementary material for: Clinical management and outcomes of acute febrile illness in children attending a tertiary hospital in southern Ethiopia
Source: BMC Infect Dis. 2022 May 4;22:434. doi: 10.1186/s12879-022-07424-0 (PMC9069758; doi:10.1186/s12879-022-07424-0)
Supplement: Supplementary file 6 — Additional file 6: Table S6. Predictors of overprescribing antimalarials on initial management to children without confirmed malaria at HUCSH, 2018-2019 [file 12879_2022_7424_MOESM6_ESM.docx]

S6 Table: Predictors of overprescribing antimalarials on initial management to children without confirmed malaria at HUCSH, 2018-2019

| Characteristics | Antimalarial prescription | | COR (95% CI)  (Included in the analysis, N=411) | AOR (95% CI) |
| --- | --- | --- | --- | --- |
|  | **Not prescribed n (%) N= 381** | **Overprescribed n (%) N= 30** |  |  |
| Residence Adm. Region |  |  |  |  |
| SNNPR-Hawassa | 219 (57.5) | 21 (70.0) | 1 | - |
| SNNPR-other | 54 (14.2) | 5 (16.7) | 0.97 (0.35-2.68) |  |
| Oromia | 108 (28.3) | 4 (13.3) | 0.39 (0.13-1.15) |  |
| Gender |  |  |  |  |
| Male | 228 (59.8) | 12 (40.0) | 1 | 1 |
| Female | 153 (40.2) | 18 (60.0) | **2.24 (1.05-4.77)*** | 1.98 (0.84-4.68) |
| Age |  |  |  |  |
| 2 – 11 m | 124 (32.5) | 2 (6.7) | 1 | 1 |
| 12 – 35 m | 131 (34.4) | 12 (40.0) | **5.68 (1.23-25.9)*** | 4.12 (0.83-20.4) |
| 36 – 59 m | 62 (16.3) | 11 (36.7) | **11.0 (2.37-51.2)*** | **11.3 (2.24-57.4)*** |
| 5 – 12 y | 64 (16.8) | 5 (16.7) | 4.84 (0.91-25.7) | 4.01 (0.69-23.4) |
| Duration of fever |  |  |  |  |
| 1 day | 103 (27.0) | 7 (23.3) | 1 | - |
| 2 – 4 days | 208 (54.6) | 18 (60.0) | 1.27 (0.52-3.15) |  |
| 5 – 7 days | 70 (18.4) | 5 (16.7) | 1.05 (0.32-3.45) |  |
| Antibacterial treatment prior to visit for current episode |  |  |  |  |
| Yes | 99 (26.0) | 3 (10.0) | 0.32 (0.09-1.07) | - |
| No | 282 (74.0) | 27 (90.0) | 1 |  |
| Axillary temperature |  |  |  |  |
| <37.5ºC ^§^ | 41 (10.8) | 1 (3.3) | **0.08 (0.01-0.60)*** | **0.05 (0.01-0.46)*** |
| 37.5 – 38.9ºC | 300 (78.7) | 16 (53.3) | **0.16 (0.07-0.37)*** | **0.19 (0.08-0.48)*** |
| ≥39ºC | 40 (10.5) | 13 (43.3) | 1 | 1 |
| Cough |  |  |  |  |
| Yes | 217 (57.0) | 8 (26.7) | **0.28 (0.12-0.63)*** | **0.29 (0.12-0.72)*** |
| No | 164 (43.0) | 22 (73.3) | 1 | 1 |
| Headache |  |  |  |  |
| Yes | 16 (12.6)^d^ | 4 (25.0)^e^ | 2.31 (0.66-8.05) | - |
| No | 111 (87.4)^d^ | 12 (75.0)^e^ | 1^Ꞣ^ |  |
| Vomiting |  |  |  |  |
| Yes | 137 (36.0) | 14 (46.7) | 1.56 (0.74-3.29) | - |
| No | 244 (64.0) | 16 (53.3) | 1 |  |
| Diarrhoea |  |  |  |  |
| Yes | 74 (19.4) | 3 (10.0) | 0.46 (0.14-1.56) | - |
| No | 307 (80.6) | 27 (90.0) | 1 |  |
| Tachypnea |  |  |  |  |
| Yes | 216 (56.7) | 16 (53.3) | 0.87 (0.41-1.84) | - |
| No | 165 (43.3) | 14 (46.7) | 1 |  |

| Tachycardia |  |  |  |  |
| --- | --- | --- | --- | --- |
| Yes | 145 (38.1) | 12 (40.0) | 1.09 (0.51-2.32) | - |
| No | 236 (61.9) | 18 (60.0) | 1 |  |
| Lower chest indrawing/ retraction |  |  |  |  |
| Yes | 80 (21.0) | 3 (10.0) | 0.42 (0.12-1.41) | - |
| No | 301 (79.0) | 27 (90.0) | 1 |  |
| Crepitation |  |  |  |  |
| Yes | 100 (26.2) | 5 (16.7) | 0.56 (0.21-1.51) | - |
| No | 281 (73.8) | 25 (83.3) | 1 |  |
| WBC count |  |  |  |  |
| Normal | 281 (74.7)^f^ | 20 (66.7) | 1^Ꞧ^ | 1 |
| High | 64 (17.0)^f^ | 2 (6.7) | 0.44 (0.10-1.93) | 0.41 (0.09-1.94) |
| Low | 31 (8.2)^f^ | 8 (26.7) | **3.63 (1.47-8.92)*** | 2.29 (0.78-6.73) |
| Anaemia |  |  |  |  |
| Yes | 40 (10.6)^f^ | 8 (26.7) | **3.06 (1.28-7.31)*** | **3.45 (1.20-9.89)*** |
| No | 336 (89.4)^f^ | 22 (73.3) | 1^Ꞧ^ | 1 |
| WAZ |  |  |  |  |
| Normal (≥ -2) | 277 (74.9)^g^ | 22 (78.6)^h^ | 1^Ɬ^ | - |
| Underweight (< -2) | 93 (25.1)^g^ | 6 (21.4)^h^ | 0.81 (0.32-2.07) |  |
| HAZ |  |  |  |  |
| Normal (≥ -2) | 299 (78.7)^k^ | 25 (83.3) | 1^Ꝑ^ | - |
| Stunting (< -2) | 81 (21.3)^k^ | 5 (16.7) | 0.74 (0.27-1.99) |  |
| BMI-AZ |  |  |  |  |
| Normal (≥ -2) | 273 (71.8)^k^ | 21 (70.0) | 1^Ꝑ^ | - |
| Wasting (< -2) | 107 (28.2)^k^ | 9 (30.0) | 1.09 (0.49-2.46) |  |

SNNPR, Southern Nations and Nationalities Peoples’ Region, COR, crude odds ratio, AOR, adjusted odds ratio, WBC, white blood cell; WAZ, weight-for-age z-score; HAZ, height-for-age z-score; BMI-AZ, body-mass-index-for-age z-score; m, month; y, years

^d^(N=127); ^e^(N=16); ^f^(N=376); ^g^(N= 370); ^h^(N= 28); ^k^(N= 380)

Included in the analysis; ^Ꞣ^(N=143); ^Ꞧ^(N=406); ^Ɬ^(N=398); ^Ꝑ^(N=410)

* Significantly associated (p-value < 0.05)

**^§^** History of fever episode at least once in the preceding 48 hours
