# Supplementary material for: Clinical management and outcomes of acute febrile illness in children attending a tertiary hospital in southern Ethiopia
Source: BMC Infect Dis. 2022 May 4;22:434. doi: 10.1186/s12879-022-07424-0 (PMC9069758; doi:10.1186/s12879-022-07424-0)
Supplement: Supplementary file 7 — Additional file 7: Table S7. Predictors of persisting fever or death by day 7 (±1) among children with pneumonia at HUCSH, 2018-2019. [file 12879_2022_7424_MOESM7_ESM.docx]

S7 Table: Predictors of persisting fever or death by day 7 (±1) among children with pneumonia at HUCSH, 2018-2019

| Characteristics | Pneumonia cases | | COR (95% CI)  (Included in the analysis, N=168) | AOR (95% CI) |
| --- | --- | --- | --- | --- |
|  | **Resolved fever n (%) N= 143** | **Persisted fever/**  **Died**  **n (%) N= 25** |  |  |
| Residence Adm. Region |  |  |  |  |
| SNNPR-Hawassa | 75 (52.4) | 8 (32.0) | 1 | 1 |
| SNNPR-other | 19 (13.3) | 7 (28.0) | **3.45 (1.11-10.7)*** | 2.85 (0.71-11.4) |
| Oromia | 49 (34.3) | 10 (40.0) | 1.91 (0.71-5.19) | 1.34 (0.40-4.45) |
| Gender |  |  |  |  |
| Male | 78 (54.5) | 17 (68.0) | 1.77 (0.72-4.37) | - |
| Female | 65 (45.5) | 8 (32.0) | 1 |  |
| Age |  |  |  |  |
| 2 – 11 m | 49 (34.3) | 18 (72.0) | 1 | 1 |
| 12 – 35 m | 60 (42.0) | 5 (20.0) | **0.23 (0.08-0.66)*** | **0.16 (0.03-0.76)*** |
| ≥ 36 m | 34 (23.8) | 2 (8.0) | **0.16 (0.04-0.74)*** | **0.07 (0.01-0.76)*** |
| Duration of fever |  |  |  |  |
| 1 day | 28 (19.6) | 3 (12.0) | 1 |  |
| 2-4 days | 82 (57.3) | 16 (64.0) | 1.82 (0.49-6.72) | - |
| 5-7 days | 33 (23.1) | 6 (24.0) | 1.70 (0.39-7.41) |  |
| Antibacterial treatment prior to visit for current episode |  |  |  |  |
| Yes | 41 (28.7) | 9 (36.0) | 1.40 (0.57-3.42) | - |
| No | 102 (71.3) | 16 (64.0) | 1 |  |
| Vaccination status |  |  |  |  |
| Completed | 101 (70.6) | 9 (36.0) | 1 | 1 |
| Vaccinated for age | 35 (24.5) | 13 (52.0) | **4.17 (1.64-10.9)*** | 1.18 (0.29-4.78) |
| Other | 7 (4.9) | 3 (12.0) | **4.81 (1.06-21.9)*** | 5.40 (0.79-37.1) |
| Axillary temperature |  |  |  |  |
| <37.5ºC ^§^ | 11 (7.7) | 1 (4.0) | 0.21 (0.02-1.94) | 0.47 (0.04-5.55) |
| 37.5 – 38.9ºC | 116 (81.1) | 17 (68.0) | **0.34 (0.12-0.93)*** | 0.60 (0.15-2.33) |
| ≥39ºC | 16 (11.2) | 7 (28.0) | 1 | 1 |
| Vomiting |  |  |  |  |
| Yes | 53 (37.1) | 4 (16.0) | **0.32 (0.11-0.99)*** | **0.21 (0.05-0.79)*** |
| No | 90 (62.9) | 21 (84.0) | 1 | 1 |
| Diarrhoea |  |  |  |  |
| Yes | 20 (14.0) | 3 (12.0) | 0.84 (0.23-3.06) | - |
| No | 123 (86.0) | 22 (88.0) | 1 |  |
| Grunting |  |  |  |  |
| Yes | 27 (18.9) | 4 (16.0) | 0.82 (0.26-2.58) | - |
| No | 116 (81.1) | 21 (84.0) | 1 |  |
| Tachypnea |  |  |  |  |
| Yes | 122 (85.3) | 23 (92.0) | 1.98 (0.43-9.03) | - |
| No | 21 (14.7) | 2 (8.0) | 1 |  |

| Tachycardia |  |  |  |  |
| --- | --- | --- | --- | --- |
| Yes | 65 (45.5) | 13 (52.0) | 1.30 (0.56-3.04) | - |
| No | 78 (54.5) | 12 (48.0) | 1 |  |
| Lower chest indrawing/ retraction |  |  |  |  |
| Yes | 59 (41.3) | 15 (60.0) | 2.14 (0.90-5.08) | - |
| No | 84 (58.7) | 10 (40.0) | 1 |  |
| Crepitation |  |  |  |  |
| Yes | 79 (55.2) | 17 (68.0) | 1.72 (0.70-4.25) | - |
| No | 64 (44.8) | 8 (32.0) | 1 |  |
| WBC count |  |  |  |  |
| Normal | 104 (73.2)^m^ | 15 (60.0) | 1^₩^ | - |
| High | 27 (19.0)^m^ | 8 (32.0) | 2.05 (0.79-5.35) |  |
| Low | 11 (7.7)^m^ | 2 (8.0) | 1.26 (0.25-6.25) |  |
| Anaemia |  |  |  |  |
| Yes | 13 (9.2)^m^ | 3 (12.0) | 1.35 (0.36-5.14) | - |
| No | 129 (90.8)^m^ | 22 (88.0) | 1^₩^ |  |
| Urinary tract infection |  |  |  |  |
| Yes | 28 (20.9)^p^ | 8 (33.3)^q^ | 1.89 (0.74-4.87) | - |
| No | 106 (79.1)^p^ | 16 (66.7)^q^ | 1^₮^ |  |
| Bacteraemia/candidaemia |  |  |  |  |
| Yes | 11 (7.9)^r^ | 4 (16.0) | 2.23 (0.65-7.67) | - |
| No | 129 (92.1)^r^ | 21 (84.0) | 1^¢^ |  |
| WAZ |  |  |  |  |
| Normal (≥ -2) | 105 (73.9)^m^ | 12 (50.0)^q^ | 1^¥^ | 1 |
| Underweight (< -2) | 37 (26.1)^m^ | 12 (50.0)^q^ | **2.84 (1.17-6.87)*** | **3.63 (1.14-11.6)*** |
| HAZ |  |  |  |  |
| Normal (≥ -2) | 109 (76.2) | 17 (68.0) | 1 | - |
| Stunting (< -2) | 34 (23.8) | 8 (32.0) | 1.51 (0.60-3.80) |  |
| BMI-AZ |  |  |  |  |
| Normal (≥ -2) | 102 (71.3) | 15 (60.0) | 1 | - |
| Wasting (< -2) | 41 (28.7) | 10 (40.0) | 1.66 (0.69-3.99) |  |

SNNPR, Southern Nations and Nationalities Peoples’ Region, COR, crude odds ratio, AOR, adjusted odds ratio, WBC, white blood cell; WAZ, weight-for-age z-score; HAZ, height-for-age z-score; BMI-AZ, body-mass-index-for-age z-score; m, month; y, years

^m^(N=142); ^p^(N=134); ^q^(N=24); ^r^(N=140)

Included in the analysis; ^₩^(N=167); ^₮^(N=158); ^¢^(N=165); ^¥^(N=166)

* Significantly associated (p-value < 0.05)

**^§^** History of fever episode at least once in the preceding 48 hours
